# The genomic landscape of metastatic clear cell renal cell carcinoma after systemic therapy

**DOI:** 10.1002/1878-0261.13204

**Published:** 2022-04-05

**Authors:** Johannes C. van der Mijn, Kenneth W. Eng, Pooja Chandra, Evan Fernandez, Sinan Ramazanoglu, Alexandros Sigaras, Clara Oromendia, Lorraine J. Gudas, Scott T. Tagawa, David M. Nanus, Bishoy F. Faltas, Himisha Beltran, Cora N. Sternberg, Olivier Elemento, Andrea Sboner, Juan Miguel Mosquera, Ana M. Molina

**Affiliations:** ^1^ 12295 Department of Pharmacology Weill Cornell Medicine New York NY USA; ^2^ Department of Medical Oncology The Netherlands Cancer Institute (NKI) Amsterdam The Netherlands; ^3^ 12295 Englander Institute for Precision Medicine Weill Cornell Medicine New York NY USA; ^4^ 12295 Institute for Computational Biomedicine Weill Cornell Medicine New York NY USA; ^5^ 12295 Department of Physiology and Biophysics Weill Cornell Medicine New York NY USA; ^6^ 12295 Division of Hematology/Oncology Department of Medicine Weill Cornell Medicine New York NY USA; ^7^ 12295 Department of Pathology and Laboratory Medicine Weill Cornell Medicine New York NY USA

**Keywords:** cancer, genomics, immunotherapy, kidney, metastasis, VEGF

## Abstract

Primary clear cell renal cell carcinoma (ccRCC) has been previously characterized, but the genomic landscape of metastatic ccRCC is largely unexplored. Here, we performed whole exome sequencing (WES) in 68 samples from 44 patients with ccRCC, including 52 samples from a metastatic site. *SETD2*, *PBRM1*, *APC* and *VHL* were the most frequently mutated genes in the metastatic ccRCC cohort. *RBM10* and *FBXW7* were also among the 10 most frequently mutated genes in metastatic tissues. Recurrent somatic copy number variations (CNV) were observed at the previously identified regions 3p25, 9p21 and 14q25, but also at 6p21 (*CDKN1A*) and 13q14 (*RB1)*. No statistically significant differences were found between samples from therapy‐naïve and pretreated patients. Clonal evolution analyses with multiple samples from 13 patients suggested that early appearance of CNVs at 3p25, 9p21 and 14q25 may be associated with rapid clinical progression. Overall, the genomic landscapes of primary and metastatic ccRCC seem to share frequent CNVs at 3p25, 9p21 and 14q25. Future work will clarify the implication of *RBM10* and *FBXW7* mutations and 6p21 and 13q14 CNVs in metastatic ccRCC.

## Graphical abstract

Molecular analysis of lesions that drive progression of kidney cancer has been performed with primary kidney tumours. In this manuscript, we analysed genomic alterations present in various metastatic lesions. We discovered two genes and chromosomal locations that are frequently altered, particularly in metastatic lesions, as well as a pattern of alterations that might be associated with poor prognosis.

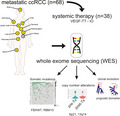

**Abbreviations**
ccRCC: clear cell renal cell carcinoma
CNV: copy number variations
EIPM: Englander Institute for Precision Medicine
Indel: insertion–deletion mutation
PBMC: peripheral blood mononuclear cells
SCNA: somatic copy number alterations
SNV: single nucleotide variant
TMB: tumour mutational burden
VAF: variant allele frequency
VEGF‐TT: vascular endothelial growth factor‐targeted therapy
WCM: Weill Cornell Medicine
WES: whole exome sequencing

## 1 Introduction

Clear cell renal cell carcinoma (ccRCC) is the most common histological subtype of kidney cancer in adults. Molecular profiling studies of primary kidney tumours have uncovered numerous genetic alterations [1, 2, 3, 4, 5], identifying recurrent loss of function mutations in the genes *VHL*, *PBRM1*, *BAP1* and *SETD2* as critical drivers of carcinogenesis. While these large‐scale genomics studies revealed a relatively low tumour mutational burden, they noted a proportionally large fraction of the genome affected by somatic copy number alterations [1]. Particularly, regions located at the chromosomal arms 3p, 14q, 9p and 5q have repeatedly been found to be altered in primary tumours of patients with ccRCC [6, 7, 8, 9].

Previous research has compared the genomic profiles of primary tumours with metastatic sites by targeted next‐generation sequencing. These studies showed comparable frequencies of mutations in genes such as *VHL*, *PBRM1, BAP1* or *SETD2* in these samples [10, 11]. A study using multiregional sequencing of paired primary and metastatic tumours revealed enrichment of clones with copy number losses at 9p and 14q in metastatic compared with primary kidney tumours [8]. This finding is in line with previous studies performed by the TCGA which suggested that loss of the *CDKN2A* locus at 9p21 in primary ccRCC tumours predicts an inferior prognosis [1]. Subclonal diversification and early branching evolution were identified as favourable prognostic molecular events, while early clonal fixation of multiple presumed molecular driver events, such as loss of 9p and/or 14q, is associated with rapid clinical progression [8].

Numerous systemic therapies are available for patients with metastatic ccRCC and include vascular endothelial growth factor (VEGF)‐targeted therapy and immune checkpoint inhibitors. In the majority of patients, these treatments induce a response or stability of disease but ultimately patients progress on therapy [12, 13]. The degree by which systemic therapy alters the genomic landscape in ccRCC is not well defined. Here, we performed a systematic survey of genomic alterations in a cohort of patients with metastatic ccRCC treated with systemic therapy. Our analysis includes the following: (a) somatic mutations and copy number alterations detected through whole exome sequencing (WES) from a wide range of metastatic sites and (b) comparison of genomic profiles of samples that were collected before and after systemic therapy. In 13 cases, multiple samples were collected from different anatomic locations, allowing for analysis of molecular evolutionary patterns.

## 2 Materials and methods

### 2.1 Clinical cohort

All patients analysed in this study consented to participation in the Englander Institute for Precision Medicine (EIPM) study at Weill Cornell Medicine (WCM). The study, that aimed to analyse the molecular alterations underlying the disease of patients with advanced cancer, was approved by the local institutional review board (IRB 1305013903) [14]. All experiments presented in this manuscript were undertaken after written informed consent of each subject. The study methodologies conformed to the standards set by the Declaration of Helsinki. Records of patients diagnosed with ccRCC were retrospectively reviewed. From each individual patient, the demographic, pathological and clinical characteristics as specified at their first contact at the Hematology/Oncology department were recorded. The clinical disease course, including time of histological diagnosis, local treatment history (surgery, radiation) and responses to various systemic therapies as provided to the patient according to routine clinical practice were registered. Prognostic classification at the time of diagnosis was performed according to the previously reported criteria of the IMDC [15]. Two patients provided written informed consent prior to death for a rapid autopsy in order to perform post‐mortem multisite sampling and were reported previously [16].

### 2.2 Sample processing and whole exome sequencing (WES)

We used fresh‐frozen tumour tissue biopsies (*n* = 41) or FFPE tumour samples (*n* = 27) and peripheral blood mononuclear cells (PBMC) for the analysis. We confirmed the presence of sufficient ccRCC tumour tissue in each tumour tissue biopsy through histological analysis of H&E‐stained slides by genitourinary pathologists (B.D.R., J.M.M.). Tumour purity was estimated through CLONET, which determines tumour tissue abundance based on the analysis of 334 single nucleotide polymorphisms (SNPs) in paired normal and tumour tissue samples and confirmed this assessment by pathological review [17]. Subsequently, tumour and germline DNA was extracted using Promega Maxwell 16 MDx DNA purification kits. All paired tumour and normal samples that yielded ≥200 ng DNA of sufficient quality, as confirmed by real‐time PCR, were used for WES analysis. DNA libraries were prepared using the Agilent Haloplex Exome system for targeted gene capture (21 522 genes) followed by next‐generation sequencing (NGS) on an Illumina HiSeq 2500 platform (2x150BP) [18].

### 2.3 Data processing and quality control

All samples were analysed through the Exome Cancer Test v1.0 (EXaCT‐1.0) bioinformatics pipeline [14, 18]. We assessed the quality of the raw reads by FastQC (https://www.bioinformatics.babraham.ac.uk/projects/fastqc/ last accessed 12/27/2019). All computational analyses were performed using the Weill Cornell Medicine high‐performance computing cluster (HPC). Short reads were aligned to GRC37/hg19 reference using Burrows‐Wheelers Aligner (BWA) [19]. The coverage was calculated by computing the average number of reads found overlapping a region of the Agilent HaloPlex Exome kit (https://www.agilent.com/cs/library/usermanuals/ public/G9906‐90000_HaloPlexExome_Manual.pdf Last accessed 12/27/2019) (reference 80‐100x). The quality of the alignment was determined by calculation of the per cent mapped reads overlapping any capture region of the kit (reference 85–95%) and the total number of mapped reads of each sample.

### 2.4 Somatic mutation analyses

Somatic mutations were identified in tumour samples through our in‐house SNVseeqer pipeline [20]. To correct for expression levels and gene size and allow comparison with TCGA data, we applied the MutSigCV algorithm [21]. Point mutations found in the matching normal sample were filtered out as normal polymorphisms. Non‐synonymous point mutations that cause amino acid changes, according to SNVseeqer, were kept. GATK somatic indel was used with default parameters to detect insertions and deletions (indels) in samples. Each somatic mutation required a minimum of at least 10 aligned reads for inclusion. The variant allele frequency (VAF) was corrected based on the CLONET tumour content estimation [17]. Variants with a corrected VAF lower than 10% were filtered out. As an additional quality control, frequently reported somatic mutations were interrogated by COSMIC, using samtools [22]. Each SNV that was reported in COSMIC at least 10 times and had a corrected VAF > 5% in the tumour and < 1% in the normal sample was kept. The tumour mutational burden (TMB) was calculated as the total number of non‐synonymous mutated bases in the tumour genome divided by the Mb of genome covered.

### 2.5 Somatic copy number analyses

To detect somatic copy number alterations (SCNAs), the normalized relative coverage of capture regions in tumour and normal samples was calculated. Capture regions with a total coverage < 100 reads in both tumour and normal sample were excluded from the analysis. The read counts of individual regions were normalized to the total number of reads in each individual sample after correction for tumour purity as determined by CLONET [17]. Ratio of the normalized read counts in the tumour sample to the normalized read counts in normal sample was calculated and log2 transformed. The values obtained after normalization were segmented using the Circular Binary Segmentation algorithm implemented in the R package DNAcopy to group the regions with similar log2 to values into segments and the average log2 value was assigned to the whole segment. The segments were ordered karyotypically and sorted by genomic coordinates. Altered genomic segments were annotated to chromosomal arm and cytobands. We used a log2 threshold of 0.5 for a DNA copy number gain or loss. Segments with log2 > 1 are considered amplified, log2 between 0.5 and 1 are considered to have copy number gain. Log2 value < −1 means deletion and log2 between −0.5 and −1 is copy number loss. All samples that showed genome‐wide copy number gains/losses were manually reviewed to determine the likelihood of ploidy differences and exclude errors in the normalization process.

### 2.6 Phylogenetic analysis

High fidelity SNVs were used to generate phylogenetic trees. We implemented CLONET to compute the clonality of SNVs in absence or presence of altered copy number segments [17]. In the phylogenetic trees, each node represents an individual tumour sample and is connected to other nodes by an edge. The length of an edge is proportional to the number of SNVs, with the least common ancestor located as the most distant node. A branch represents a time point in the evolution of the tumour where two distinct cell populations emerged. The length of the branches models the number of SNVs that are private to each node. No formal bootstrapping analysis or statistical tests were performed; the analysis should be interpreted as a qualitative analysis.

## 3 Results

### 3.1 Demographic and clinical characteristics

Forty‐four patients with ccRCC consented to participate in the EIPM study and were successfully analysed by WES. The demographic and clinical characteristics are summarized in Table 1. The majority consisted of white non‐hispanic males (*n* = 28 and *n* = 36, respectively) with a median age of 65 years old (range 38–86 years). Each individual patient was classified according to the previously defined IMDC prognostic criteria [15]. The majority (52%) of the patients was classified as intermediate risk, while 32% and 16% were favourable or poor risk, respectively. Twenty‐one patients (47%) presented with metastatic disease. All the remaining patients, except for patient WCMC‐RCC‐010, developed metastatic disease during follow‐up. Most patients had radiographic evidence of metastatic sites in the lungs (88%), liver (36%), bone (50%), lymph nodes (59%) or brain (32%).

**Table 1:** Patient characteristics.

	Patient number (*n* = 44)
Age	
Median, years (range)	65 (38–86)
Gender, *N* (%)	
Male	36 (82)
Female	8 (18)
Race/ethnicity, *N* (%)	
White non‐hispanic	28 (64)
Black non‐hispanic	0
Hispanic	3 (7)
Other/unknown	13 (30)
IMDC prognostic score, *N* (%)	
Favourable	14 (32)
Intermediate	23 (52)
Poor	7 (16)
Metastatic sites, *N* (%)	
Lung	39 (88)
Liver	16 (36)
Bone	22 (50)
Lymph nodes	26 (59)
Brain	14 (32)

**Fig. 1:**
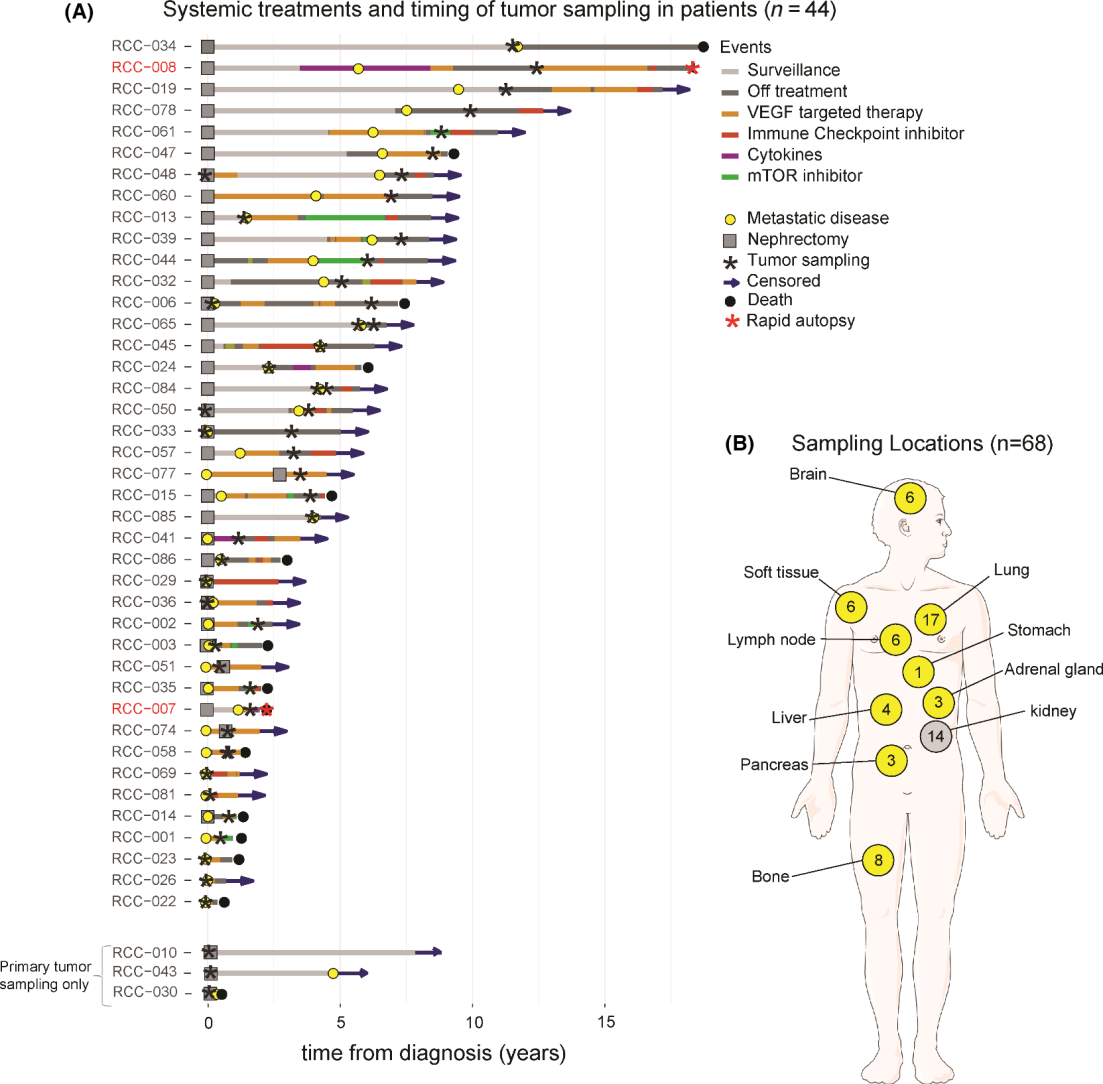
Disease course, treatments and timing of tumour sampling in patients with metastatic clear cell renal cell carcinoma (ccRCC) at Weill Cornell Medicine (WCM) are shown in A. The disease sites that were sampled for analysis by whole exome sequencing (B). In total, 44 patients received treatment for ccRCC at WCM and underwent tumour tissue sampling for molecular profiling (A). In two patients (labelled red), we performed a rapid autopsy for collection of tissue from multiple disease sites in parallel. The Swimmers plot illustrates that most samples were collected in patients with advanced disease and frequently sampled after treatment with systemic therapy (A). In addition to 14 primary kidney tumours, the majority of samples was derived from various metastatic disease sites (B).

The various therapies are summarized in Table 2 and illustrated in Fig. 1A. The majority of the patients had a nephrectomy (*n* = 37, 84%) and received systemic therapy (*n* = 39, 89%). Palliative radiotherapy was provided to 17 patients (39%). VEGF‐targeted therapy was the most frequently provided systemic therapy, followed by immune checkpoint inhibitor treatment. Pazopanib (*n* = 23, 52%) was the most commonly prescribed agent, followed by sunitinib (*n* = 15), axitinib (*n* = 11), cabozantinib (*n* = 8) or other agents (*n* = 15). In total, 20 patients received more than one line of therapy with VEGF‐targeted therapy. From the immune checkpoint inhibitors, nivolumab was the most frequently prescribed agent (*n* = 21), followed by ipilimumab (*n* = 5) and pembrolizumab (*n* = 3). Other systemic therapies that were initiated included mTOR inhibitors (everolimus, temsirolimus), cytokines (interferon gamma, interleukin 2) and various other systemic agents. In total, 13 patients (30%) received combination therapy, which most frequently consisted of nivolumab/ipilimumab (*n* = 5) or everolimus/lenvatinib (*n* = 3).

**Table 2:** Focal and systemic therapies provided for kidney cancer.

	Clear cell RCC (*n* = 44)
Nephrectomy, *N* (%)	37 (84)
Radiotherapy, *N* (%)	17 (39)
Systemic therapy, *N* (%)	36 (82)
VEGF‐targeted therapy, *N* (%)	
Pazopanib	23 (52)
Sunitinib	15 (34)
Axitinib	11 (25)
Cabozantinib	8 (18)
Other	15 (34)
Immune checkpoint inhibitor, *N* (%)	
Nivolumab	21 (48)
Ipilimumab	5 (11)
Pembrolizumab	3 (7)
mTOR inhibitors, *N* (%)	18 (41)
Cytokines/other, *N* (%)	12 (27)

### 3.2 Timing and location of tumour sampling in individual ccRCC patients

In total, 68 ccRCC tissue samples from 44 patients were successfully sequenced. The various anatomic sites that were sampled in our cohort are illustrated in Fig. 1B. In 41 patients, the samples were derived from various metastatic sites, including the lung (*n* = 17), bone (*n* = 7), lymph nodes (*n* = 6), brain (*n* = 6) and soft tissue (*n* = 6). In three cases, only the primary kidney tumours were sampled for sequencing, while in 11 cases, paired primary and metastatic tumour samples were available. Twenty‐one patients were diagnosed with metastatic ccRCC, while 23 patients initially presented with localized ccRCC and developed metastatic disease after a median of 3.7 years (range 0.2–11.7 years). Sixteen patients eventually died of metastatic ccRCC after a median follow‐up of 4.6 years (range 0.4–18.5 years), while 25 patients were still actively followed. Three patients were lost to follow‐up. Thirty‐nine patients received systemic therapy in their disease course, with VEGF‐targeted therapy (*n* = 36), immune checkpoint inhibitors (*n* = 23) and mTOR inhibitors (*n* = 18) as most common therapies. The median progression‐free survival (best response in case of multiple agents from the same class) to these treatments was 11.0 months (range 1.0–53.5 months), 4.8 months (range 1.4–28.8) and 2.7 months (range 0.7–35.7 months), respectively.

### 3.3 The somatic mutational landscape of metastatic ccRCC after systemic therapy

**Fig. 2:**
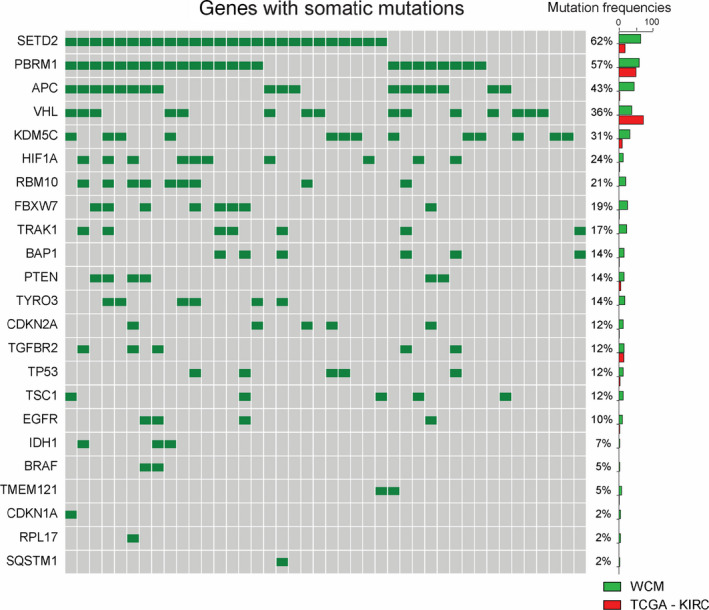
The most frequent somatic mutations in our cohort of metastatic clear cell renal cell carcinoma (ccRCC). We performed whole exome sequencing in 43 patients with metastatic ccRCC and 1 patient with localized ccRCC and determined the most frequently mutated genes in our cohort. Individual patients are presented in the columns (grey squares) with presence of a somatic mutations indicated by green squares. All paired tumour tissue and peripheral blood mononuclear cell (PBMC) samples were analysed by the EXaCT v1.0 bioinformatics pipeline. Somatic mutations were recovered by application of SNVseeqer. To correct for expression levels and gene size, we applied the MutSigCV algorithm. The respective mutation frequencies in the TCGA cohort (KIRC) and WCM cohort are illustrated by the bar graph on the right hand side. *SETD2, PBRM1, APC*, *VHL*, *KDM5C, HIF1A, RBM10 and FBXW7* were the most frequently mutated genes in our cohort. Statistical significance was assessed by using the Mann–Whitney–Wilcoxon test. We did not detect any statistically significant differences in the mutation frequencies in the TCGA‐KIRC and WCM datasets.

We performed WES on 68 tumour tissue samples from 43 patients with metastatic ccRCC and from 1 patient who did not develop metastatic disease. Figure 2 shows the most frequently mutated genes in our cohort of patients. To correct for expression levels and gene size, we applied the MutSigCV algorithm [21]. The tumour suppressor genes *SETD2* and *PBRM1* were the most frequently mutated genes in our cohort (mutated in 62% and 57% of the patients, respectively). Other frequently mutated genes were *APC* (43%), *VHL* (36%), *KDM5C* (31%) and *HIF1A* (24%). While the previous genes are frequently part of targeted sequencing panels, we also identified mutations in *RBM10* (21%) and *FBXW7* (19%) in multiple cases. The majority of the somatic mutations were missense mutations, but particularly *SETD2*, *PBRM1*, *VHL* and *KDM5C* contained a higher proportion of potentially functionally relevant nonsense and frameshift insertions or deletions (indels). To determine whether these mutational frequencies deviate from previous studies, we compared our results (WCM) with the somatic mutational landscape from the TCGA ccRCC study (KIRC, Fig. 2). The predominantly metastatic tumours in our cohort contained more frequent mutations in several genes such as *SETD2*, *APC*, *KDM5C, HIF1A, RBM10, TRAK1* and *FBXW7*, while we detected lower mutation rates in *VHL* compared to the primary tumours analysed in the TCGA study. None of these differences reached statistical significance in our analysis.

**Fig. 3:**
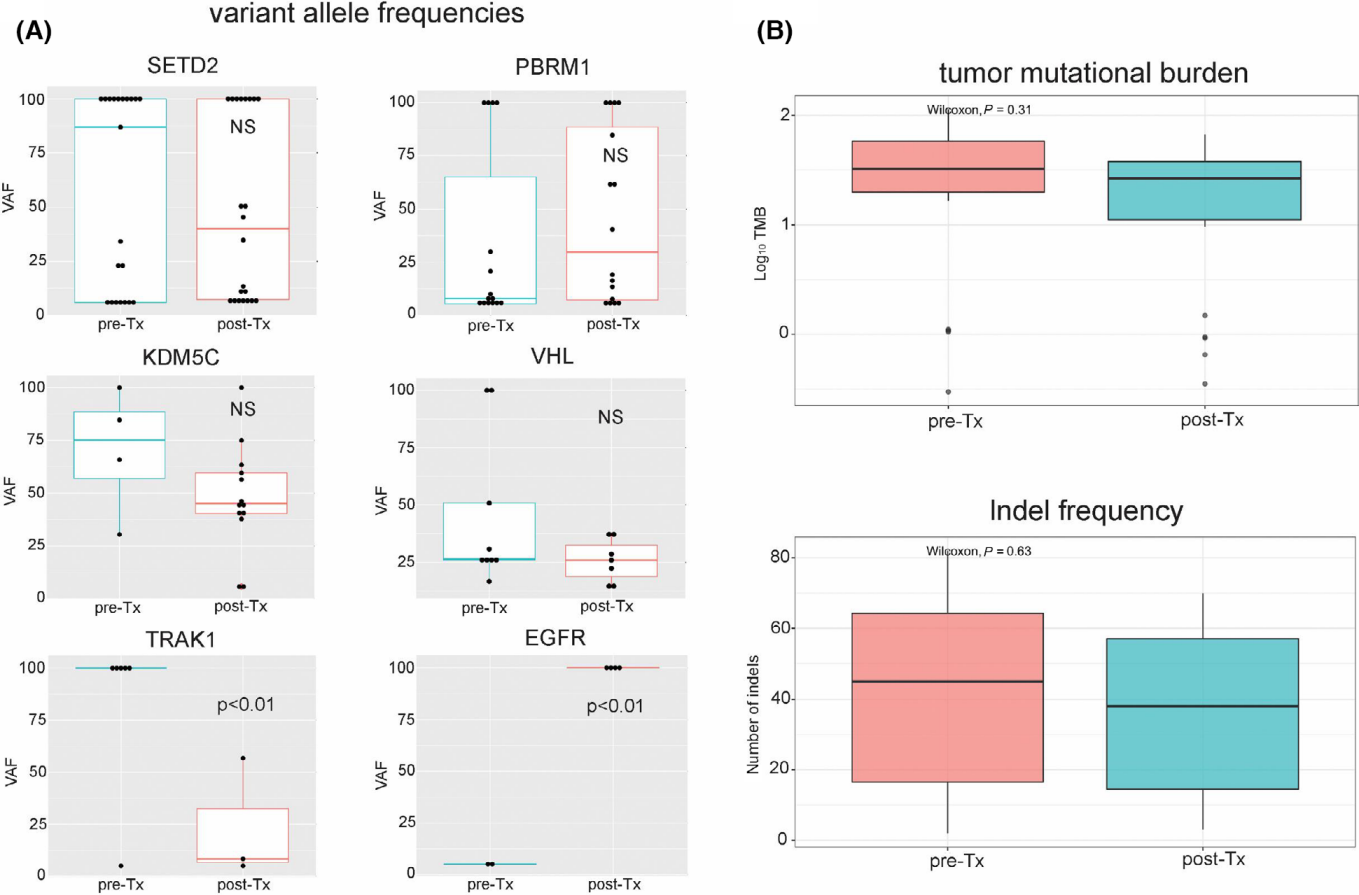
The impact of systemic therapy on the variant allele frequency (VAF) of frequent somatic mutations, tumour mutational burden (TMB) and insertion/deletion (indel) frequencies. In total, 68 tumour samples were analysed. 38 were sampled after systemic treatment, while 30 samples were derived from therapy‐naïve ccRCC tumours. In five patients, paired samples before and after systemic therapy were available. We compared the VAF of all frequently mutated genes, TMB and indel frequency to determine whether therapy induces clonal selection in tumours. We did not detect statistically significant differences in the VAF in *SETD2*, *PBRM1*, *KDM5C* and *VHL* between therapy‐naïve and pretreated tumour samples. Systemic treatment induced a decreased VAF of mutations in *TRAK1* and increased VAF in *EGFR* (A). Boxplots are shown with standard error; statistical significance was assessed by using the Mann–Whitney–Wilcoxon test. We did not detect any statistically significant differences in TMB or indel frequencies (B).

From the 68 tumour samples, 38 were sampled after patients received systemic treatment, while 30 samples were derived from therapy‐naïve patients. Two patients received only cytokines prior to tumour sampling, while others were pretreated with VEGF‐targeted therapy (*n* = 36), mTOR inhibitors (*n* = 15) or immune checkpoint inhibitors (*n* = 7). The majority of the pretreated patients were exposed to multiple treatment lines (24/38, 63%). We compared the mutational frequencies between samples obtained pre‐ and post‐therapy. After correction for multiple testing, we detected no statistically significant enrichment of mutated genes in any of the groups. To investigate whether certain mutations are involved in therapy resistance and are therefore enriched in samples collected after therapy, we analysed the variant allele frequency (VAF) of the most frequently mutated genes. We detected no statistically significant differences in the VAF of *SETD2, PBRM1, VHL* and *KDM5C* between therapy‐naïve and pretreated samples (Fig. 3A). Among the other frequently mutated genes, we observed a significant depletion of *TRAK1* mutations (*P* < 0.01), while *EGFR* mutations displayed a higher VAF (*P* < 0.01) in samples collected after therapy (Fig. 3A). *TRAK1* and *EGFR* mutations were present in 9 and 6 tumours from 7 and 4 patients, respectively. We observed no statistically significant differences in tumour mutational burden (TMB) and indel frequency between therapy‐naïve and pretreated samples (Fig. 3B). In summary, these results indicate that systemic therapy had a minor impact on the somatic mutational landscape of metastatic ccRCC and suggest that systemic therapy potentially induces selection of clones that contain *EGFR* mutations.

### 3.4 Somatic copy number alterations in metastatic ccRCC after systemic therapy

**Fig. 4:**
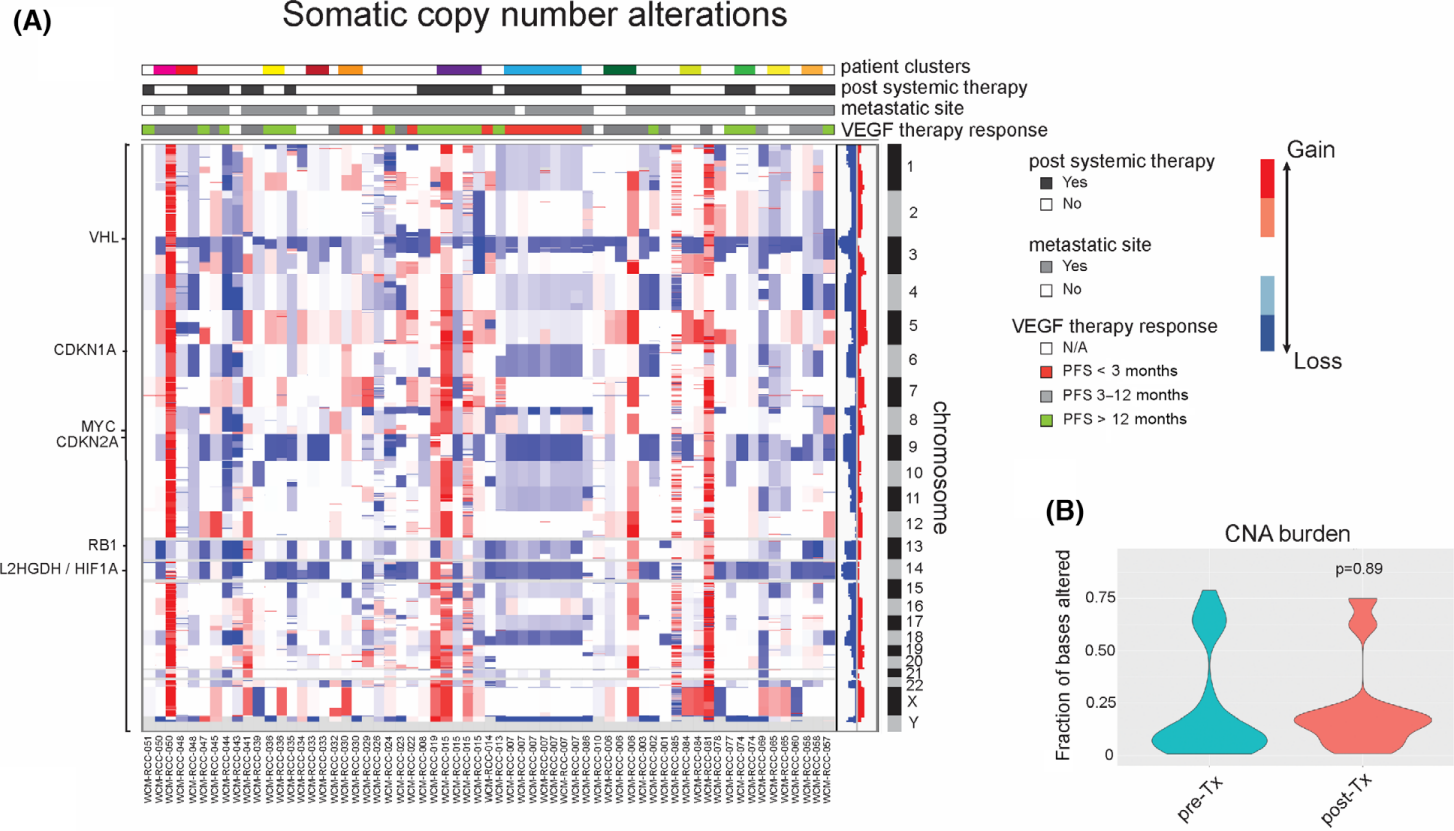
The most frequent somatic copy number alterations in metastatic clear cell renal cell carcinoma (ccRCC). To detect genome‐wide somatic copy number alterations (SCNAs), we calculated the relative coverage of capture regions in tumour and normal samples (*n* = 63). The read counts of individual regions were normalized to the total number of reads in each individual sample after correction for tumour purity as determined by CLONET. Panel A shows a supervised clustering of samples according to patient ID with all different samples from individual patients represented by unique colours. We also annotated the samples with exposure to prior systemic therapy, sampling form a metastatic site and response to the most commonly provided treatment (VEGF targeted therapy). We observed recurrent somatic copy number deletions at 3p25, 9p21, 14q25, 6p21 and 13q14. Genes located in the deleted regions are depicted on the left. To determine whether systemic therapy influences the prevalence of SCNAs, we calculated the copy number alteration (CNA) burden. Statistical significance was assessed by using the Mann–Whitney–Wilcoxon test. No statistically significant differences were present in CNA burden between therapy‐naïve and pretreated samples (B).

The global pattern of somatic copy number alterations in our cohort of metastatic ccRCC is shown in Fig. 4. We detected frequent copy number losses at 3p25 (*n* = 36 patients), 9p21 (*n* = 30) and 14q25 (*n* = 17). We detected additional deletions located at chromosome 6 (*n* = 18) and 13 (*n* = 23), which included the regions 6p21 and 13q14 in several cases (Fig. 4A). Interestingly, these regions contain the well‐known tumour suppressor genes *RB1* and *CDKN1A* (p21^waf^), which are both involved in cell cycle progression. Similar to previous studies [1, 6], we detected frequent amplifications at chromosomal arm 5q in our cohort (*n* = 21). We compared the prevalence of SCNAs in therapy naïve and pretreated tumour samples. The pretreated tumour samples contained an increased frequency of deletions at chromosome 6, although not statistically significant (*P* = 0.11). The overall SCNA burden was similar between the therapy‐naïve and pretreated groups (Fig. 4B). We observed no statistically significant differences in the frequency of deletions at 3p25, 9p21, 14q25 and 13q14 between the treatment groups. In conclusion, our results show additional chromosomal regions with frequent copy number losses at 6p21 and 13q14 in metastatic ccRCC tumours.

### 3.5 Clonal evolution in selected cases with a distinct clinical disease course

**Fig. 5:**
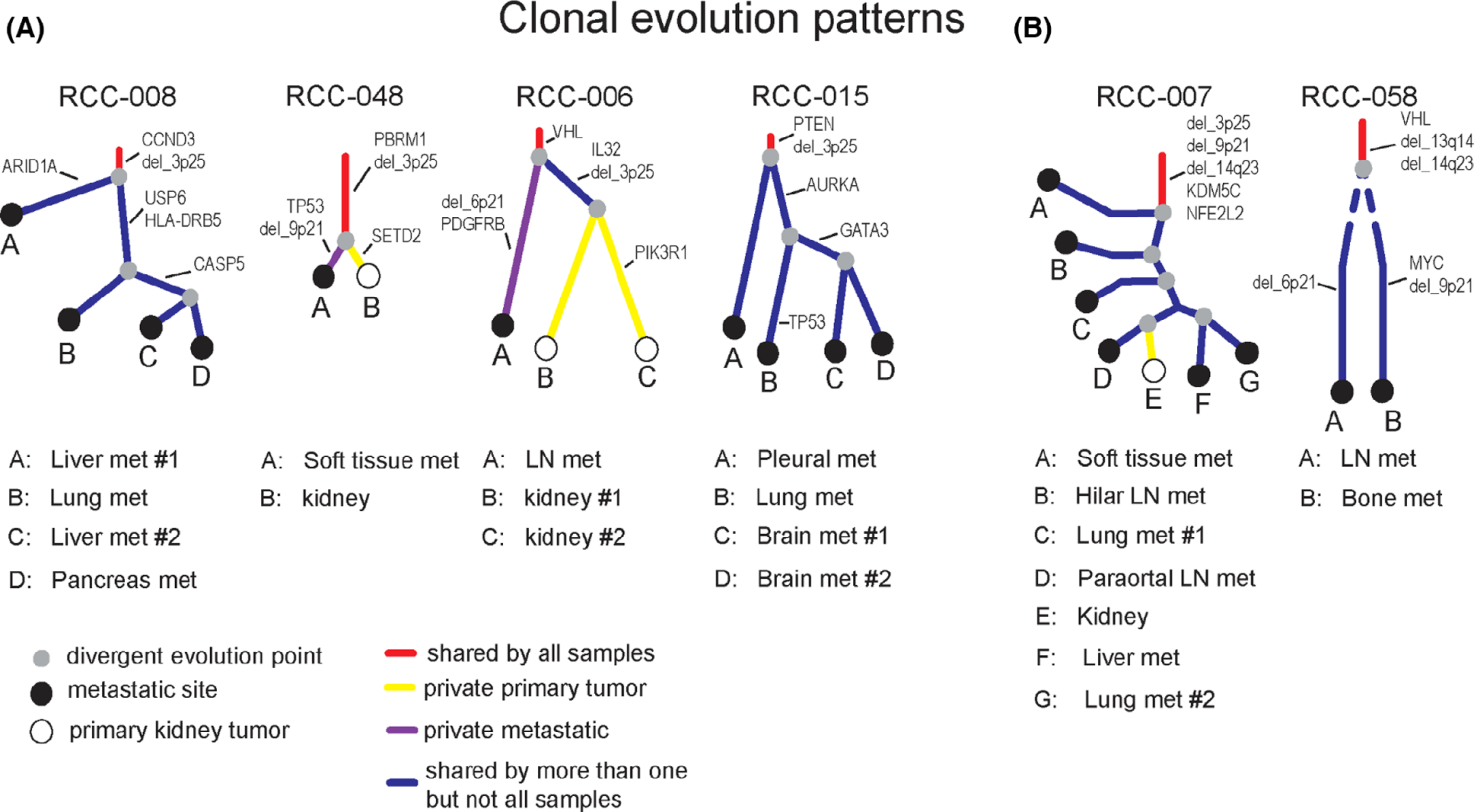
Molecular evolution in cases with a distinct disease course. In four cases with an indolent disease course (RCC‐008, RCC‐048, RCC‐006, RCC‐015, A) and two cases with rapid clinical disease progression (RCC‐007, RCC‐058, B), multiple tumour samples were analysed. Phylogenetic trees were constructed based on variant allele frequencies (VAFs) of individual somatic mutations and corrected for read‐depth due to copy number alterations. This analysis illustrates subclonal diversification with relatively few copy number alterations in patients with indolent disease course, versus early clonal fixation of multiple presumed driver events in patients with rapid disease progression.

We collected multiple tumour samples in 13 patients with metastatic ccRCC, allowing for analysis of clonal evolution of the disease. Two of these patients (RCC‐007, RCC‐058) had widespread metastatic disease with rapid clinical progression and death within 2 years of diagnosis. In contrast, four patients (RCC‐008, RCC‐048, RCC‐006, RCC‐015) had an indolent disease course with a relatively long overall survival with metastatic ccRCC (4.4–18.3 years). To determine whether these two groups display different evolutionary patterns, we constructed phylogenetic trees based on VAFs of individual somatic mutations and corrected for read‐depth due to SCNAs (Fig. 5). All cases showed loss‐of‐function mutation or copy number loss of the *VHL* locus in the trunk of the phylogenetic tree.

In two of the cases with an indolent disease course (RCC‐006, RCC‐048, Fig. 5A) we analysed the primary tumour and a metastatic site with 6.0 and 6.4 years between sampling. In the other two cases with slowly progressive disease (RCC‐008, RCC‐015, Fig. 5A), several metastatic sites were sampled shortly before or after death. RCC‐006, RCC‐048 and RCC‐008 showed a relatively low SCNA burden, while RCC‐015 showed various subclonal copy number alterations that differed between metastatic sites. Most of these tumours appeared to be driven, at least in part, by individual pathogenic mutations, for example, in *CCND3* (p.S259A) in RCC‐008, and two different *PTEN* mutations (p.C136F, p.P95R) in RCC‐015. RCC‐006 appeared to have acquired metastatic fitness through selection of a clone with copy number loss at chromosome 6 in combination with a pathogenic mutation in *PDGFRB* (p.R853W), while metastasis in RCC‐048 evolved through development of copy number loss at 9p in combination with a novel *TP53* mutation (p.D241).

Both patients with rapid progression (RCC‐007, RCC‐058, Fig. 5B) showed a molecular profile consistent with early clonal fixation of multiple copy number events, including loss of regions at 3p25, 14q25 and either 13q14 (RCC‐058) or 9p21 (RCC‐007) in all tumour samples. Collectively, these results confirm previous observations that rapid disease progression is associated with early presence of multiple clonal drivers, while an attenuated disease course is associated with branched evolution with subclonal diversification.

## 4 Discussion

Previous molecular profiling studies identified the most common genetic alterations in primary tumours of patients with ccRCC [1, 2, 3, 5, 8]. Here, we performed genome‐wide profiling of specimens from patients with metastatic ccRCC. Previous research in a similarly sized cohort of patients with paired primary and matched metastatic tumour samples, identified somatic copy number losses at 9p21 (*CDKN2A*) and 14q (*L2HGDH*, *HIF1A*) as critical molecular alterations towards the development of metastatic disease in patients with ccRCC [5]. Our analysis confirms that these regions are indeed subject of frequent molecular alterations and demonstrates additional somatic copy number losses at 6p21 and 13q14 in metastatic ccRCC tumours. Loss of the tumour suppressor genes *RB1* (6p21) and *CDKN1A* (13q14) may explain enrichment of these genetic alterations in our cohort of metastatic tumours. Both of these molecules prevent, similar to *CDKN2A*, cell cycle progression through their interaction with cyclins and cyclin‐dependent kinases (CDKs) [23]. Loss of the chromosomal regions may promote cell cycle progression, clonal expansion, and metastasis. Our analysis also yielded *RBM10* and *FBXW7* as frequently mutated genes in metastatic ccRCC tumours. *FBXW7* acts as a substrate recognition unit of an E3 ubiquitin protein ligase and has been identified as a tumour suppressor gene in other cancers [24]. Previous research revealed that loss‐of‐function mutations in *FBXW7* in melanoma tumours confer resistance to anti‐PD1 therapies, which are currently frontline treatment in ccRCC [25]. RBM10 is an RNA binding protein known to be a TFEB fusion partner in MIT translocation‐associated RCC [26]. Somatic mutations in RBM10 have also been detected in NSCLC tumours and were found to be associated with increased influx of CD8+ T cells and IFN‐G transcriptional signatures [27]. Therefore, RBM10 and FBXW7 mutations may act as a biomarker in patients with ccRCC receiving immune checkpoint inhibitors. We believe that validation of these findings is required in a larger sample cohort.

We evaluated the impact of systemic therapy and analysed evolutionary patterns in tumours from patients with paired samples. Previous retrospective cohort studies identified potential predictive value of *PBRM1*, *BAP1* and *TP53* mutations in primary tumour tissue from ccRCC patients that received treatment with first‐line tyrosine kinase inhibitors [28, 29]. An earlier study found a similar pattern with potential durable responses to VEGF‐targeted therapy in patients with *PBRM1* mutations, while *TP53* mutations were associated with primary refractory disease [30]. These results have not yet been confirmed in prospective clinical trials, but suggest that these mutations are potential determinants of the response to VEGF‐targeted therapy. In our analysis, none of the most frequently mutated genes, including *VHL*, *PBRM1*, *BAP1* and *KDM5C*, showed increased mutation rates or changes in VAF in the samples collected after therapy. The lack of clonal selection, indicated by the comparable VAF of the most frequent molecular alterations after treatment, suggests a limited role for these molecular alterations in therapy resistance. In contrast, samples from patients that received systemic therapy prior to tumour sampling did show higher VAF of *EGFR* mutations, which were detected in four patients (10%) in our cohort, and a non‐statistically significant enrichment of chromosome 6 copy number deletions. Previous research in patients with non‐small cell lung cancer (NSCLC) showed a reduction in the EGFR mutation frequency after chemotherapy [31], but no reports have been published about the impact of targeted therapy or immune checkpoint inhibitors. Our results suggest that VEGF‐targeted therapy and/or PD‐1 blockade in contrast could induce selection of subclones that are driven by *EGFR* signalling. These results could lead to design of novel treatment strategies with for example *EGFR* tyrosine kinase inhibitors. Indeed, a previous study in which ccRCC patients were treated with the EGFR inhibitor erlotinib in combination with bevacizumab demonstrated clinical activity in selected patients [32]. A limitation of our study is the number samples that were collected after immune checkpoint inhibitors, which are now the mainstay in the treatment of ccRCC. Two recent WES studies included either only samples collected prior the immune checkpoint inhibitor therapy or focused on immune cell infiltrates after treatment [33, 34]. One study analysed ccRCC samples from thirteen patients before and after anti‐PD‐1 treatment with WES [35]. No specific genomic events were described to be enriched or depleted specifically after therapy. Additional prospective research is warranted in larger sample cohorts before conclusions can be drawn about the role of these molecular alterations in disease progression. In a limited number of patients, paired tumour samples from different metastatic sites were available for clonal evolution studies. Such multiregional sequencing strategies from small numbers of patients have provided critical insights in tumour heterogeneity and evolutionary patterns in the past [36, 37]. This analysis confirmed that subclonal diversification is associated with an indolent disease course, while early clonal fixation of multiple molecular driver events predisposes to rapid clinical progression. These results build on previous research from the TRACERx consortium, which used a cohort of 101 patients with ccRCC [8]. Limitations of our study are the small number of paired samples before and after systemic therapy, as well as the variety of sampling locations and variable windows between sample collections. While the variety of sampling locations increased the sample heterogeneity, we did not identify clustering according to metastatic site, indicating that organ tropism of cancer cells is not likely driven by individual genomic alterations. Previous preclinical research has suggested epigenetic alterations as potential determinant of multiorgan metastasis, which is a finding that warrants clinical validation [38]. Nonetheless, these results provide a comprehensive overview of the genomic alterations that contribute to the development of metastatic ccRCC.

## 5 Conclusion

We performed whole exome sequencing of metastatic ccRCC specimens. The genomic landscapes of primary and metastatic ccRCC seem to share frequent CNVs at 3p25, 9p21 and 14q25. Future work will clarify the implication of *RBM10* and *FBXW7* mutations and 6p21 and 13q14 CNVs in metastatic ccRCC.

## Conflict of interest

HB has served as consultant/advisory board member for Janssen, Sanofi Genzyme, Astellas, Astra Zeneca, Merck, Pfizer, Foundation Medicine, and Blue Earth and has received research funding from Janssen Oncology (inst), AbbVie/Stemcentrx (Inst), Eli Lilly (Inst), Millennium Pharmaceuticals (Inst). BF has served as consultant for QED therapeutics and advisory board member for Merck, Immunomedics/Gilead and QED therapeutics, has received patent royalties from Immunomedics/Gilead, honoraria from Urotoday, research support from Eli‐Lilly. CS reports consulting for Janssen‐Cilag, Astellas Pharma, Sanofi‐Genzyme, Bayer, Pfizer, Merck, MSD, AstraZeneca, Clovis, Immunomedics/Gilead, BMS, Foundation Medicine, UroToday, Medscape, and the NCI; institutional funding from Cougar Biotechnology/Janssen, Medivation/Pfizer, Clovis Oncology and Roche‐Genentech. AM reports consulting for Eisai, Exelixis and Janssen.

### Peer review

The peer review history for this article is available at https://publons.com/publon/10.1002/1878‐0261.13204.

## Author contributions

JvdM, KE, PC, EF, SR, AS, CO, AS, JMM, AM were involved in conducting research, software development, formal analysis, data curation, validation and visualization; ST, DN, BF, HB, CS, JMM, AM provided samples, assisted in interpretation of results and supervised research, LG, DN, OE, AS, HB, BF were involved in development of methodologies, provided resources and funding; JvdM, LG, DN, AS, JMM and AM wrote the original draft; all authors reviewed, edited and approved the final version of the manuscript.

## Data Availability

The cBioPortal cohort associated with this manuscript (id: ccrcc_wcm_2022) contains samples annotated with clinical information as registered for the individual patient upon first contact with their treating physician at our hospital. All samples with sufficient data quality of both complete somatic mutation and somatic copy number analysis were included.
